# Should patients undergoing hematopoietic stem cell transplantation undergo screening and monitoring for skin cancer?^☆^

**DOI:** 10.1016/j.abd.2024.06.007

**Published:** 2025-01-10

**Authors:** Miguel Mansilla-Polo

**Affiliations:** a Department of Dermatology, Hospital Universitario y Politécnico La Fe, Valencia, Spain; b Instituto de Investigación Sanitaria (IIS) La Fe, Valencia, Spain; c Universitat de València, Valencia, Spain, Dermatology, Valencia, Spain

*Dear Editor,*

In recent years, it has become evident that solid organ transplant (SOT) recipients exhibit a higher incidence of cutaneous cancers (CC) compared to the general population. This phenomenon is particularly favored by the chronic immunosuppression they undergo to prevent organ transplant rejection. In fact, a predictive tool for cutaneous cancer in SOT patients has gained popularity in recent years, known as SUNTRAC® (skin and ultraviolet neoplasia transplant risk assessment calculator), which utilizes five items: race, age at transplant, history of cutaneous cancer prior to transplant, gender, and abdominal or thoracic transplant location.^1^ This tool has recently been validated.^2^

In the case of hematopoietic stem cell transplantation (HSCT), recent studies, based on the potential elevated incidence of CC, have advocated for the need for screening and monitoring of CC in HSCT patients.^3^ We present two clinical cases of patients who developed two cutaneous squamous cell carcinomas (cSCC) following HSCT and had a grim outcome leading to the demise of both.

The first case (Fig. 1A‒1B) involved a 54-year-old male, recipient of allogeneic HSCT (allo-HSCT) for chronic myeloid leukemia three years prior, who presented with an cSCC on the lower lip. He was initially treated with surgery and clean margins, but after 3 months of follow-up, he developed regional lymph node, pulmonary and bone metastases, confirmed by ultrasound-guided biopsy, transbronchial biopsy, and CT-guided biopsy, respectively. The lesions progressed rapidly, and palliative treatment was chosen, leading to his death within 6 months. The second case (Fig. 2A‒2B) involved a 63-year-old male, recipient of autologous HSCT (auto-HSCT) for multiple myeloma five years prior, who presented with a cSCC on the left commissure of the lips. At diagnosis, he had regional lymph node and (confirmed by ultrasound-guided biopsy) pulmonary metastases (confirmed by transbronchial biopsy) and a severe pleural effusion. Due to his poor baseline condition (ECOG3) and at the explicit request of the patient and his family, palliative treatment was chosen, which resulted in his death less than 3 months after diagnosis. Neither of the two patients had additional risk factors beyond those inherent to HSCT. Specifically, in the first patient, the use of voriconazole as prophylaxis against fungal infection and chronic graft versus host disease (GVHD) with only oral involvement (untreated at the time of CC diagnosis, previously treated with topical corticosteroids) were identified as possible risk factors for cSCC. In the second patient, total body irradiation and chronic GVHD with oral and cutaneous involvement (untreated at the time of diagnosis of CHD, previously treated with topical corticosteroids, systemic corticosteroids, and topical calcineurin inhibitors) were identified as possible risk factors for cSCC.

**Figure 1:**
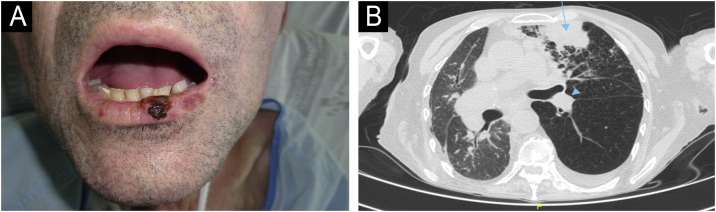
**Patient Number 1.** In Patient 1, a cutaneous squamous cell carcinoma (cSCC) measuring 1.3 × 1 cm is observed on the lower lip (A). The axial computed tomography (CT) scan (B) reveals a mass consistent with pulmonary metastasis in the anterior segment of the upper left lobe (blue arrow), along with an associated left paratracheal metastasis (blue arrowheads).

**Figure 2:**
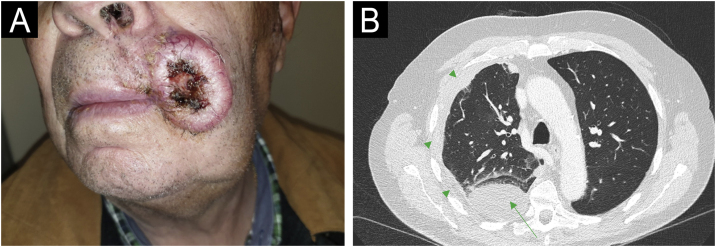
**Patient Number 2.** For Patient 2, a cSCC measuring 4.2 × 3.5 cm is observed at the left commissure of the lips (A). The axial CT scan (B) displays a mass consistent with pulmonary metastasis in the posterior segment of the lower right lobe (green arrows), accompanied by pleural effusion (green arrowheads) and associated laminar atelectasis.

In recent years, a higher incidence of CC has been postulated in HSCT patients.^3^ This may be attributed to intrinsic factors related to HSCT, such as immunosuppression derived from pre-HSCT conditioning, allo-HSCT, medications used for graft-versus-host disease prophylaxis (especially azathioprine or cyclosporine), or exposure to voriconazole as antifungal prophylaxis. Intrinsic factors in the HSCT recipient, such as age, history of sun damage, skin type, patient's gender, or personal history of CC, could also contribute to this risk. The risk may be higher for keratinocytic tumors (basal cell carcinoma or cSCC) but has also been described for melanoma.^4^, ^5^

Unlike SOT, there is a lack of in-depth studies evaluating the incidence, typology, and characteristics of CC post-HSCT. Similarly, there is a lack of studies assessing whether these tumors could be high-risk. This was the case for our two patients who presented with highly aggressive cSCCs that led to their demise in a short span. Through this article, it is proposed that regular dermatological surveillance and prompt recognition of precancerous and cancerous lesions in HSCT patients are crucial for prognosis and management. Prospective studies with large populations are essential to evaluate the necessity of CC screening in patients undergoing HSCT, including indications and the need for follow-up.

## Financial support

None declared.

## Author’ contributions

Miguel Mansila-Porto: Managed clinical diagnosis and procedures, and wrote the full paper.

## Conflicts of interest

None declared.
